# Supplementary material for: Phytochemicals and Glioma: Results from Dietary Mixed Exposure
Source: Brain Sci. 2023 Jun 2;13(6):902. doi: 10.3390/brainsci13060902 (PMC10296340; doi:10.3390/brainsci13060902)
Supplement: Supplementary file 1 [file brainsci-13-00902-s001.zip › brainsci-2419003-supplementary.pdf]

Supplementary material

Table S1. Correlations between dietary phytochemicals among the case-control participants.

Table S2. Adjusted ORs and 95% CIs for gliomas in subgroups.

Table S3. Adjusted ORs and 95% CIs for the association between the phytochemical intake per unit body weight and glioma.

Table S4. The condPIP of the five phytochemicals.

Figure S1. Exposure–response plots (95%CIs) for associations between log-transformed intakes of individual phytochemicals and glioma when all other phytochemicals were fixed at their median intakes.

Figure S2. Bivariate exposure-response plots for log-transformed intakes of individual phytochemicals and glioma when a second phytochemical was fixed at its 25th, 50th, or 75th percentile and the other phytochemicals were fixed at their medians.

Table S1. Correlations between dietary phytochemicals among the case-control participants.

	**Carotene**	**Flavonoid**	**Soy Isoflavone**	**Anthocyanin**	**Resveratrol**
Carotene	1.000	0.726	0.423	0.573	0.634
Flavonoid		1.000	0.435	0.622	0.815
Soy isoflavone			1.000	0.392	0.386
Anthocyanin				1.000	0.664
Resveratrol					1.000

Note: *P values* of all correlation coefficients were less than 0.01

Table S2. Adjusted ORs and 95% CIs for gliomas in subgroups.

Subgroup[a]	T1	T2	T3	*P*-*trend*
Age				
≤40(n = 500)				
Carotene	1	0.40(0.23–0.72)	0.07(0.04–0.14)	<0.001
Flavonoid	1	0.41(0.23–0.71)	0.25(0.13–0.47)	<0.001
Soy isoflavone	1	1.64(0.95–2.85)	0.32(0.17–0.58)	<0.001
Anthocyanin	1	0.66(0.38–1.16)	0.09(0.05–0.17)	<0.001
Resveratrol	1	0.89(0.51–1.54)	0.24(0.13–0.45)	<0.001
> 41(n = 512)				
Carotene	1	0.21(0.12–0.39)	0.06(0.03–0.12)	<0.001
Flavonoid	1	0.29(0.17–0.50)	0.17(0.09–0.32)	<0.001
Soy isoflavone	1	0.64(0.38–1.08)	0.24(0.14–0.42)	<0.001
Anthocyanin	1	0.23(0.12–0.42)	0.02(0.01–0.05)	<0.001
Resveratrol	1	0.40(0.23–0.69)	0.18(0.10–0.33)	<0.001
Sex				
Male(n = 568)				
Carotene	1	0.39(0.24–0.65)	0.06(0.03–0.11)	<0.001
Flavonoid	1	0.46(0.28–0.74)	0.15(0.09–0.27)	<0.001
Soy isoflavone	1	1.59(0.99–2.55)	0.39(0.24–0.65)	<0.001
Anthocyanin	1	0.52(0.31–0.87)	0.04(0.02–0.07)	<0.001
Resveratrol	1	0.87(0.54–1.40)	0.25(0.15–0.42)	<0.001
Female(n = 444)				
Carotene	1	0.43(0.23–0.81)	0.10(0.05–0.22)	<0.001
Flavonoid	1	0.35(0.19–0.67)	0.21(0.10–0.45)	<0.001
Soy isoflavone	1	0.68(0.36–1.28)	0.16(0.08–0.33)	<0.001
Anthocyanin	1	0.28(0.14–0.54)	0.06(0.03–0.12)	<0.001
Resveratrol	1	0.40(0.21–0.76)	0.25(0.13–0.49)	<0.001
BMI				
≤23.31(n = 506)				
Carotene	1	0.34(0.19–0.60)	0.10(0.05–0.20)	<0.001
Flavonoid	1	0.34(0.20–0.60)	0.30(0.16–0.58)	0.001
Soy isoflavone	1	1.13(0.65–1.96)	0.30(0.16–0.55)	<0.001
Anthocyanin	1	0.55(0.31–0.95)	0.11(0.06–0.21)	<0.001
Resveratrol	1	0.61(0.35–1.06)	0.25(0.13–0.46)	<0.001
> 23.31(n = 506)				
Carotene	1	0.38(0.21–0.69)	0.06(0.03–0.12)	<0.001
Flavonoid	1	0.42(0.24–0.73)	0.14(0.08–0.27)	<0.001
Soy isoflavone	1	1.01(0.59–1.71)	0.26(0.15–0.46)	<0.001
Anthocyanin	1	0.35(0.19–0.65)	0.02(0.01–0.05)	<0.001
Resveratrol	1	0.61(0.35–1.04)	0.18(0.10–0.32)	<0.001
Occupation				
Manual workers(n = 237)				
Carotene	1	0.43(0.16–1.17)	0.02(0.01–0.09)	<0.001

Flavonoid	1	0.67(0.27–1.66)	0.13(0.05–0.38)	<0.001
Soy isoflavone	1	1.01(0.41–2.51)	0.31(0.12–0.77)	0.004
Anthocyanin	1	0.24(0.08–0.76)	0.01(0.002–0.04)	<0.001
Resveratrol	1	0.19(0.08–0.50)	0.11(0.04–0.32)	<0.001
Mental workers(n = 571)				
Carotene	1	0.51(0.31–0.83)	0.10(0.06–0.18)	<0.001
Flavonoid	1	0.42(0.26–0.68)	0.25(0.14–0.44)	<0.001
Soy isoflavone	1	1.22(0.76–1.97)	0.22(0.13–0.39)	<0.001
Anthocyanin	1	0.66(0.41–1.06)	0.09(0.05–0.16)	<0.001
Resveratrol	1	0.86(0.54–1.38)	0.35(0.21–0.60)	<0.001
Others(n = 204)				
Carotene	1	0.21(0.06–0.75)	0.03(0.01–0.12)	<0.001
Flavonoid	1	0.11(0.03–0.38)	0.04(0.01–0.17)	<0.001
Soy isoflavone	1	0.20(0.07–0.64)	0.08(0.03–0.27)	<0.001
Anthocyanin	1	0.07(0.01–0.30)	0.01(0.001–0.03)	<0.001
Resveratrol	1	0.41(0.14–1.15)	0.13(0.04–0.41)	0.001
Education level				
Middle school and below(n = 385)[b]				
Carotene	1	0.34(0.16–0.76)	0.07(0.03–0.16)	<0.001
Flavonoid	1	0.26(0.13–0.55)	0.21(0.09–0.48)	0.001
Soy isoflavone	1	0.68(0.33–1.41)	0.23(0.11–0.48)	<0.001
Anthocyanin	1	0.24(0.09–0.59)	0.02(0.01–0.06)	<0.001
Resveratrol	1	0.34(0.16–0.71)	0.16(0.07–0.35)	<0.001
University and above(n = 627)				
Carotene	1	0.41(0.26–0.64)	0.09(0.05–0.15)	<0.001
Flavonoid	1	0.43(0.27–0.68)	0.24(0.14–0.41)	<0.001
Soy isoflavone	1	1.26(0.81–1.96)	0.26(0.16–0.44)	<0.001
Anthocyanin	1	0.57(0.36–0.89)	0.06(0.03–0.11)	<0.001
Resveratrol	1	0.77(0.49–1.20)	0.24(0.14–0.40)	<0.001
Household income				
<3,000 ¥/month(n = 141)				
Carotene	1	0.77(0.20–2.91)	0.06(0.01–0.30)	<0.001
Flavonoid	1	0.51(0.15–1.76)	0.59(0.15–2.36)	0.475
Soy isoflavone	1	0.64(0.17–2.40)	0.46(0.13–1.64)	0.246
Anthocyanin	1	0.18(0.04–0.83)	0.01(0.001–0.10)	<0.001
Resveratrol	1	0.17(0.04–0.70)	0.21(0.05–0.89)	0.115
3,000-10,000 ¥/month(n = 633)				
Carotene	1	0.37(0.22–0.64)	0.07(0.04–0.12)	<0.001
Flavonoid	1	0.45(0.28–0.74)	0.21(0.12–0.36)	<0.001
Soy isoflavone	1	1.25(0.77–2.02)	0.26(0.16–0.41)	<0.001
Anthocyanin	1	0.57(0.34–0.97)	0.07(0.04–0.12)	<0.001
Resveratrol	1	0.71(0.44–1.16)	0.24(0.14–0.40)	<0.001
>10,000 ¥/month(n = 238)				
Carotene	1	0.62(0.28–1.39)	0.12(0.04–0.38)	<0.001

Flavonoid	1	0.18(0.08–0.43)	0.10(0.03–0.32)	<0.001
Soy isoflavone	1	1.00(0.47–2.13)	0.54(0.21–1.37)	0.167
Anthocyanin	1	0.34(0.15–0.77)	0.03(0.01–0.11)	<0.001
Resveratrol	1	0.33(0.14–0.77)	0.14(0.05–0.40)	0.001
Smoking status				
Never smoking(n = 735)				
Carotene	1	0.41(0.26–0.65)	0.12(0.07–0.19)	<0.001
Flavonoid	1	0.45(0.29–0.70)	0.25(0.15–0.41)	<0.001
Soy isoflavone	1	1.10(0.71–1.70)	0.28(0.18–0.45)	<0.001
Anthocyanin	1	0.39(0.25–0.62)	0.07(0.04–0.12)	<0.001
Resveratrol	1	0.50(0.32–0.78)	0.22(0.13–0.37)	<0.001
Smoking(n = 277)[c]				
Carotene	1	0.47(0.21–1.06)	0.03(0.01–0.08)	<0.001
Flavonoid	1	0.29(0.14–0.63)	0.09(0.04–0.21)	<0.001
Soy isoflavone	1	0.95(0.46–1.97)	0.21(0.10–0.45)	<0.001
Anthocyanin	1	0.43(0.18–1.04)	0.01(0.004–0.04)	<0.001
Resveratrol	1	0.61(0.30–1.27)	0.19(0.09–0.42)	<0.001
History of allergies				
Yes(n = 113)				
Carotene	1	0.59(0.19–1.85)	0.16(0.04–0.66)	0.010
Flavonoid	1	0.35(0.11–1.15)	0.25(0.06–0.99)	0.049
Soy isoflavone	1	0.67(0.22–2.02)	0.05(0.01–0.29)	0.001
Anthocyanin	1	0.68(0.19–2.48)	0.002(0.001–0.06)	<0.001
Resveratrol	1	0.80(0.25–2.57)	0.43(0.11–1.68)	0.220
No(n = 899)				
Carotene	1	0.38(0.25–0.59)	0.06(0.04–0.10)	<0.001
Flavonoid	1	0.39(0.26–0.58)	0.18(0.11–0.28)	<0.001
Soy isoflavone	1	1.11(0.75–1.65)	0.31(0.20–0.47)	<0.001
Anthocyanin	1	0.44(0.28–0.68)	0.05(0.03–0.08)	<0.001
Resveratrol	1	0.54(0.36–0.81)	0.19(0.12–0.30)	<0.001
Family history of cancer				
Yes(n = 259)				
Carotene	1	0.47(0.21–1.03)	0.17(0.07–0.41)	<0.001
Flavonoid	1	0.62(0.29–1.36)	0.26(0.11–0.65)	0.003
Soy isoflavone	1	0.98(0.44–2.15)	0.22(0.10–0.50)	<0.001
Anthocyanin	1	0.38(0.16–0.90)	0.07(0.03–0.17)	<0.001
Resveratrol	1	1.03(0.47–2.24)	0.29(0.12–0.67)	0.001
No(n = 753)				
Carotene	1	0.38(0.25–0.59)	0.06(0.04–0.10)	<0.001
Flavonoid	1	0.39(0.26–0.58)	0.18(0.11–0.28)	<0.001
Soy isoflavone	1	1.11(0.75–1.65)	0.31(0.20–0.47)	<0.001
Anthocyanin	1	0.44(0.28–0.68)	0.05(0.03–0.08)	<0.001
Resveratrol	1	0.54(0.36–0.81)	0.19(0.12–0.30)	<0.001
Physical Activity				

Low(n = 301)				
Carotene	1	0.61(0.29–1.29)	0.53(0.23–1.23)	0.147
Flavonoid	1	0.33(0.15–0.72)	0.29(0.12–0.71)	0.008
Soy isoflavone	1	0.84(0.40–1.78)	0.60(0.27–1.34)	0.213
Anthocyanin	1	0.48(0.23–1.02)	0.06(0.02–0.19)	<0.001
Resveratrol	1	0.56(0.27–1.19)	0.32(0.13–0.81)	0.018
Moderate(n = 393)				
Carotene	1	0.32(0.17–0.58)	0.05(0.03–0.11)	<0.001
Flavonoid	1	0.41(0.23–0.73)	0.18(0.09–0.36)	<0.001
Soy isoflavone	1	1.15(0.65–2.05)	0.22(0.12–0.42)	<0.001
Anthocyanin	1	0.38(0.21–0.70)	0.04(0.02–0.09)	<0.001
Resveratrol	1	0.56(0.31–0.99)	0.21(0.11–0.40)	<0.001
Violent(n = 318)				
Carotene	1	0.66(0.25–1.74)	0.04(0.02–0.12)	<0.001
Flavonoid	1	0.22(0.09–0.54)	0.09(0.03–0.23)	<0.001
Soy isoflavone	1	0.58(0.25–1.37)	0.14(0.06–032)	<0.001
Anthocyanin	1	0.61(0.23–1.61)	0.06(0.03–0.15)	<0.001
Resveratrol	1	0.57(0.25–1.29)	0.20(0.09–0.44)	<0.001

[a.] Unconditional logistic regression model was used for subgroup analysis. Adjusted covariates and sex in model 2 (except for corresponding hierarchical variables).

[b.] Middle school and below included primary school and below and middle school.

[c.] Smoking included former smoking, and current smoking.

Table S3. Adjusted ORs and 95% CIs for the association between the phytochemical intake per unit body weight and glioma.

Phytochemicals[c]	Model 1[a]	*P*	Model 2[b]	*P*
**Total population**				
Carotene	0.96(0.95–0.97)	<0.001	0.95(0.94–0.97)	<0.001
Flavonoid	0.51(0.43–0.61)	<0.001	0.40(0.29–0.55)	<0.001
Soy isoflavone	0.63(0.55–0.73)	<0.001	0.54(0.43–0.68)	<0.001
Anthocyanin	0.30(0.24–0.38)	<0.001	0.29(0.20–0.40)	<0.001
Resveratrol	0.97(0.96–0.98)	<0.001	0.98(0.96–0.99)	0.007
**Astrocytoma**				
Carotene	0.95(0.94–0.97)	<0.001	0.90(0.85–0.96)	0.001
Flavonoid	0.55(0.38–0.78)	0.001	0.34(0.15–0.75)	0.007
Soy isoflavone	0.49(0.32–0.73)	0.001	0.23(0.08–0.63)	0.004
Anthocyanin	0.13(0.06–0.31)	<0.001	0.07(0.02–0.37)	0.001
Resveratrol	0.95(0.92–0.98)	0.002	0.93(0.88–0.99)	0.014
**Glioblastoma**				
Carotene	0.97(0.96–0.98)	<0.001	0.96(0.94–0.98)	<0.001
Flavonoid	0.53(0.42–0.68)	<0.001	0.38(0.21–0.71)	0.002
Soy isoflavone	0.61(0.48–0.77)	<0.001	0.41(0.24–0.70)	0.001
Anthocyanin	0.37(0.27–0.50)	<0.001	0.27(0.14–0.52)	<0.001
Resveratrol	0.97(0.96–0.99)	<0.001	0.98(0.95–1.01)	0.121
**Low grade**				
Carotene	0.95(0.93–0.97)	<0.001	0.87(0.80–0.95)	0.001
Flavonoid	0.59(0.41–0.84)	0.004	0.47(0.23–0.98)	0.044
Soy isoflavone	0.64(0.47–0.89)	0.008	0.76(0.46–1.24)	0.266
Anthocyanin	0.22(0.11–0.43)	<0.001	0.13(0.04–0.46)	0.002
Resveratrol	0.97(0.95–0.99)	0.013	0.98(0.95–1.01)	0.243
**High grade**				
Carotene	0.97(0.96–0.98)	<0.001	0.96(0.94–0.98)	<0.001
Flavonoid	0.49(0.40–0.62)	<0.001	0.28(0.16–0.48)	<0.001
Soy isoflavone	0.62(0.51–0.75)	<0.001	0.53(0.36–0.77)	0.001
Anthocyanin	0.31(0.23–0.41)	<0.001	0.22(0.13–0.39)	<0.001
Resveratrol	0.97(0.95–0.98)	<0.001	0.96(0.94–0.99)	0.004

[a.] Model 1: Unadjusted model.

[b.] Model 2: Adjusted for age, occupation, education level, household income, high-risk residential areas, smoking status, history of allergies, history of head trauma, family history of cancer, physical activity, and energy intake.

[c.] Carotene and resveratrol per 1 μg/kg increment every day, flavonoid per 1 mg/kg increment every day; soy isoflavone and anthocyanin per 100 μg/kg increments every day.

Table S4. The condPIP of the five phytochemicals.

Phytochemicals	CondPIP
Carotene	1.0000
Flavonoid	0.9964
Soy isoflavone	1.0000
Anthocyanin	1.0000
Resveratrol	0.9840

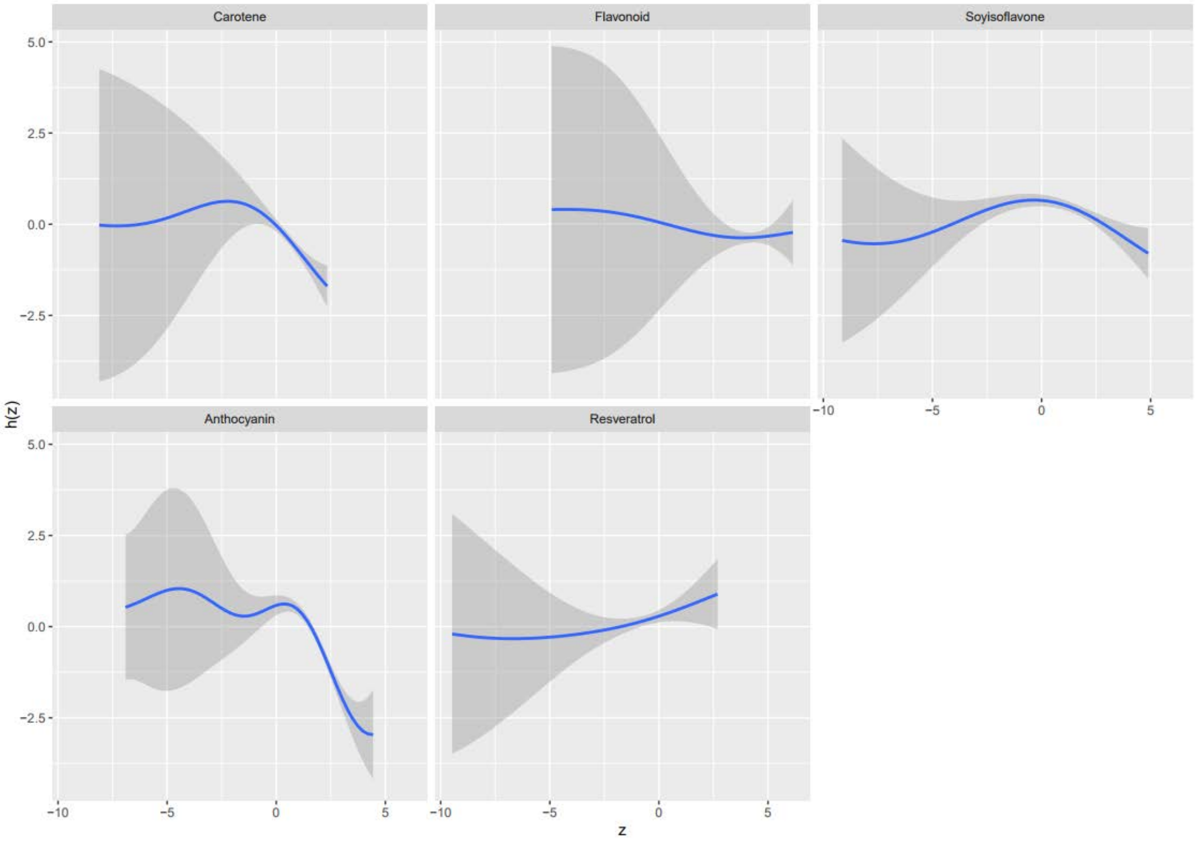

Figure S1. Exposure–response plots (95%CIs) for associations between log-transformed intakes of individual phytochemicals and glioma when all other phytochemicals were fixed at their median intakes.

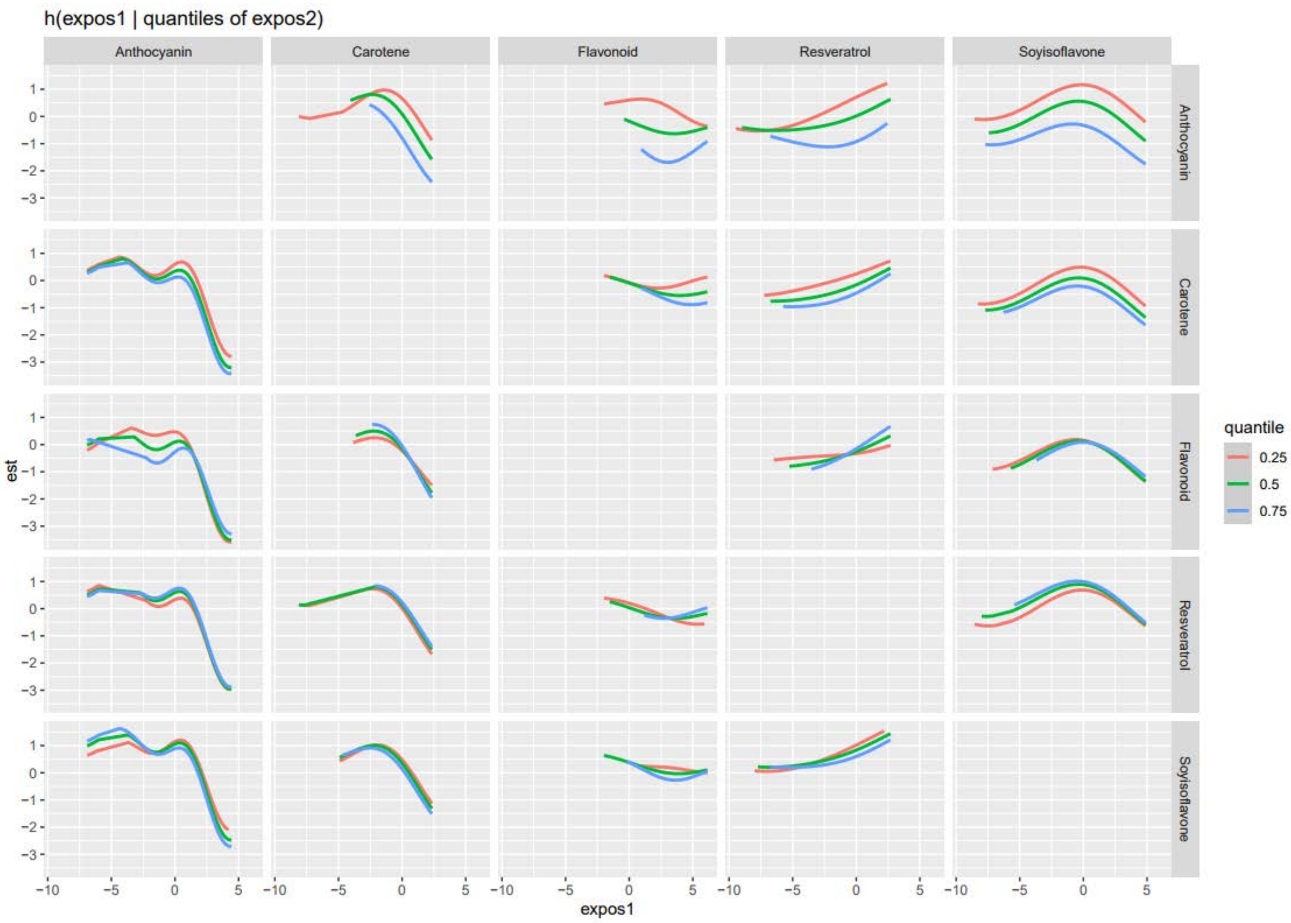

Figure S2. Bivariate exposure-response plots for log-transformed intakes of individual phytochemicals and glioma when a second phytochemical was fixed at its 25th, 50th, or 75th percentile and the other phytochemicals were fixed at their medians.
